# Highlights of the 2023 American Joint Replacement Registry Annual Report

**DOI:** 10.1016/j.artd.2024.101325

**Published:** 2024-02-29

**Authors:** Sean P. Ryan, Jeffrey B. Stambough, James I. Huddleston, Brett R. Levine

**Affiliations:** a Division of Adult Reconstruction, Duke University Medical Center, Morrisville, NC, USA; b Department of Orthopaedic Surgery, UAMS Health, Little Rock, AR, USA; c Department of Orthopaedic Surgery, Stanford University, Stanford, CA, USA; d Professor of Orthopaedics, Rush University Medical Center, Chicago, IL USA

**Keywords:** American joint replacement registry, Highlights, Total hip arthroplasty, Total knee arthroplasty

## Abstract

The 2023 report represents a full decade of published annual reports of the American Joint Replacement Registry (AJRR). The number of cases being captured continues to rapidly grow, as are over 3.2 million patients included in AJRR. Matched-pair primary and revision data is more robust with 10-year survivorship being available. Similarly, implant-specific survivorship has been included for common implants being used in the United States. The data mined from the AJRR have led to numerous publications and presentations. Numerous trends have emerged, and others have been reinforced with the most recent data. The authors encourage readers to more thoroughly review the full report at the following link: https://www.aaos.org/registries/publications/ajrr-annual-report/.

## American joint replacement registry 2023 executive summary

This year represents 10 years of published annual reports from the American Joint Replacement Registry (AJRR). Since the initial report, over 3.8 million hip and knee arthroplasty procedures have been captured from over 3.2 million patients. Integration of Medicare claims data into the AJRR has provided valuable insight into implant utilization and revision rates over the last decade. Implant-specific survivorship analyses continue to be a popular portion of the Annual Report. Matched-pair primary and revision data by implant demonstrates an overall 10-year revision rate of 2.58% for cementless total hip and 2.13% for cemented total knees. Implant-specific revision rates are available in the *Annual Report*.

Patient-reported outcome measures (PROMs) represent a highlight from this year’s *Annual Report*. As the Center for Medicare and Medicaid Services mandate for PROM collection approaches [1], AJRR has seen an expansion of reporting, with 36% of sites (496/1364) submitting PROMs. This represents a 23% increase compared to last year, and the AJRR continues to support the RegistryInsights PROM platform to facilitate greater participation in reporting. With updates to the platform made throughout the year, institutions and surgeons can view their dashboards and compare their performance to national benchmarks.

Additionally, cases performed in ambulatory surgery centers (ASCs) have continued to expand as outpatient arthroplasty procedures became more common. This year demonstrated an 84% increase in arthroplasty procedures reported by ASCs, totaling nearly 42,000 procedures in 2022. In total, the cumulative reported volume of arthroplasty procedures represents a 23% increase compared to the 2022 report, which includes a 9% increase in the number of reporting facilities. Almost 11,000 surgeons have submitted at least one procedure to the AJRR.

Quality data and reporting over the last decade has resulted in numerous publications, podiums, and poster presentations from the AJRR. Continued peer review of the data remains a focus of the organization, and recent published topics have included risk factors for revision, fracture, cement fixation, and the utilization of dual mobility articulations [[2], [3], [4], [5], [6]].

## AJRR annual report highlights

Data from the 2023 Annual Report represents 3,149,042 primary and revision hip and knee arthroplasty procedures performed from 2012-2022, with the majority of cases being primary knee (51.0%) and primary hip (33.4%) arthroplasty. For all reported procedures, the majority were female (58.5%). Total knee arthroplasty (TKA) procedures had a mean age of 67.4 years, and the average annual volume in 2022 from submitting surgeons was 56.0 cases (increased from 35.5 reported in 2022). Total hip arthroplasty (THA) procedures had a mean age of 65.4 years, and the average annual volume in 2022 from submitting surgeons was 39.2 cases (up from 27.4 reported in 2022). The most commonly reported race was non-Hispanic White (76.6%), while race remained unreported in 14.2% of cases.

Clearly, the COVID-19 pandemic created significant challenges for arthroplasty surgeons and patients. Initially, a massive decrease in case volume was reported in early 2020. However, this rebounded to normal averages just 2 months after the maximal reduction noted during the pandemic. Overall ASC volume was relatively immune to changing trends in COVID-19 incidence, as volume has slowly increased through the end of 2022. Since the pandemic, there has not been a change in the cumulative percent revision for elective THA and TKA surgeries among the Medicare population. Further, we appear to be back on track, as the overall cumulative procedural volume grew by 23% in the last year.

### Last year’s annual report highlighted several trends, which continued through 2022

• THA for femoral neck fractures has increased over the last decade, while hemiarthroplasty remains the most performed procedure.
• Postoperative length of stay continues to decrease. Primary THA procedures in 2022 had a mean length of stay of 1.2 days, compared to 3.0 days in 2012 (*P* < .0001), while THA for fracture and hemiarthroplasty length of stay has remained relatively stable. Primary TKA procedures in 2022 had a mean length of stay of 1.2 days, compared to 2.9 days in 2012 (*P* < .0001).
• 93% of primary THA and TKA patients are discharged home.
• The use of general anesthesia continues to slowly decrease, representing <40% of THA and <30% of TKA procedures.

### Trends in primary total hip arthroplasty

• For femoral neck fractures, the incidence of THA continues to increase, now representing 27.7% of the procedures. For patients <60 years of age, THA is the most common treatment for displaced femoral neck fractures.
• Cement use for femoral component fixation after a femoral neck fracture has increased over the last 5 years. Utilization is now reported in 20.74% of THA and 50.57% of hemiarthroplasty procedures. Cement is more commonly used for patients >90 years of age (52.44%), though it remains underutilized compared to international registries [[7], [8], [9], [10]].
• For elective primary THA, cemented femoral stem fixation remains limited at 4.55% of all procedures. However, the use of cemented femoral fixation has increased over time (*P* < .0001), and demonstrates a significantly lower risk for early revision due to fracture compared to cementless fixation in patients >65 years of age (hazard ratio 0.287, *P* < .0001).
• The use of dual mobility has increased over time but showed a slight decrease in 2022 (7.21% of primary THA). The shift to larger femoral heads continues with 36 mm femoral heads representing the most commonly (62.94%) used option.
• With the persistent concern of mechanically assisted crevice corrosion, ceramic femoral head use continues to increase for primary THA procedures, representing 81.43% of implanted femoral heads, while cobalt chromium (CoCr) head use continues to fall.
• Ceramic on polyethylene articulations demonstrated a corresponding increase in use (72.56%), and highly cross-linked polyethylene (95.88%) was the predominant bearing in 2022.
• Infection remains the most common reason for all-cause revision (22.51%), as well as early “linked” revision (34.84%) over the course of AJRR collection.
• Other etiologies for early revision include instability (21.9%) and periprosthetic fracture (21.4%).
• The average number of revision hip arthroplasty procedures from participating surgeons was 6.5, with 8 annual revisions placing surgeons in the 75th percentile by volume.
• 92% of patients received a meaningful improvement in their Hip Disability and Osteoarthritis Score, Joint Replacement score.

### Trends in primary total knee arthroplasty

• The use of cruciate retaining designs continues to increase, representing 56.1% of all primary TKA, while posterior stabilized utilization continues to decline.
• Patella resurfacing continues to decrease; however, it is still performed in 88.6% of primary TKA procedures (compared to 95.9% in 2012).
• Cementless fixation has substantially increased since 2012 (20.5% from 1.9%, *P* < .0001). Additionally, this includes an ∼2% increase from the 2021 report.
• Cementless fixation was associated with a reduced revision rate in men but demonstrated an increased revision rate in women >65 years of age.
• This increase in cementless fixation has been similarly demonstrated in the Swedish Arthroplasty Register [7].
• Robotic assistance has increased over 6-fold in the last 6 years, representing 13.4% of primary TKA procedures.
• The use of conventional polyethylene continues to decrease, while antioxidant polyethylene is increasing. Highly cross-linked polyethylene is the most commonly utilized material and has remained stable at about 44% of primary TKA.
• Medial and lateral unicompartmental arthroplasty procedures, as well as patellofemoral arthroplasty, have been relatively steady over the last 5 years, representing 4.0% and 0.3% of all primary knee arthroplasty procedures, respectively.
• 86% of patients received a meaningful improvement in their Knee Osteoarthritis Outcome Score, Joint Replacement score.

The scientific community has continued to improve hip and knee arthroplasty care and device surveillance due to the data provided by the AJRR, with numerous peer-reviewed publications [[2], [3], [4], [5], [6]] and poster presentations this year. The data contributed by participating institutions is critical to the success and longevity of the registry. The last decade has seen tremendous expansion in the reporting of hip and knee arthroplasty procedures throughout the United States. To participate as a contributing center, review collected data elements, or submit proposals for data analysis, visit https://www.aaos.org/registries/registry-program/american-joint-replacement-registry/.

## Conflicts of interest

S. Ryan is a paid consultant for DePuy Synthes, Zimmer Biomet, and Total Joint Orthopaedics; has stock options is Azra Care; and receives research support from Zimmer Biomet and Smith and Nephew. J. Huddleston is a paid consultant and receives royalties from Exactech and DePuy; has stock options in Corin and Porosteon; receives research support from Apple and ZimmerBiomet; receives financial/material support from WoltersKluwer; and is a board/committee member of the American Academy of Orthopaedic Surgeons (AAOS), American Joint Replacement Registry (AJRR), American Association of Hip and Knee Surgeons (AAHKS), Knee Society, and Hip Society. J. Stambough receives royalties from Signature Orthopaedics; is a paid consultant for Smith & Nephew; is an editorial board member of Journal of Arthroplasty; and is a board/committee member of American Association of Hip & Knee Surgeons (AAHKS), Education Committee AJRR, and Steering Committee. B.R. Levine is a consultate for Zimmer-Biomet, Link Medical, Enovis, and Merete. He receives royalties from Link Medical, WoltersKluwer, Slack, Inc, and Elsevier. He is on the Mid-American Orthopaedic Association Education Committee and the American Academy of Orthopaedic Surgeons Evidence-Based Quality and Value Committee and is the Orthpaedic Learning Center member of the AAOS Board of Directors and is on the Digital Media Committee of the Hip Society/Knee Society. He is the American Association of Hip and Knee Surgeons Member-at-Large and is a member of the Evidence-Based Medicine Committee. He also serves as the Deputy Editor of Arthroplasty Today.

For full disclosure statements refer to https://doi.org/10.1016/j.artd.2024.101325.

## CRediT authorship contribution statement

**Sean P. Ryan:** Project administration, Writing – original draft, Writing – review & editing. **Jeffrey B. Stambough:** Writing – original draft, Writing – review & editing. **James I. Huddleston:** Conceptualization, Writing – original draft, Writing – review & editing. **Brett R. Levine:** Supervision, Writing – original draft, Writing – review & editing.

## Appendix A Supplementary data

Conflict of Interest Statement for Huddleston

Conflict of Interest Statement for Levine

Conflict of Interest Statement for Stambough

Conflict of Interest Statement for Ryan
