# Supplementary material for: Highlights of the 2023 American Joint Replacement Registry Annual Report
Source: Arthroplast Today. 2024 Feb 29;26:101325. doi: 10.1016/j.artd.2024.101325 (PMC11239969; doi:10.1016/j.artd.2024.101325)
Supplement: Conflict of Interest Statement for Huddleston [file mmc1.docx]

# CONFLICT OF INTEREST STATEMENT

***American Association of Hip and Knee Surgeons***

(Adopted from the American Academy of Orthopaedic Surgeons disclosure statement)

The following form **must be filled out completely and submitted by each author (example, 6 authors, 6 forms).**

**All items require a response. If there is no relevant disclosure for a given item, enter "*None*.”**

Manuscript Title:

**Highlights of the 2023 American Joint Replacement Registry Annual Report**

1. Royalties from a company or supplier (The following conflicts were disclosed). Exactech, DePuy

2. Speakers bureau/paid presentations for a company or supplier (The following conflicts were disclosed) none

3A. Paid employee for a company or supplier (The following conflicts were disclosed) none

3B. Paid consultant for a company or supplier (The following conflicts were disclosed). Exactech, Depuy

3C. Unpaid consultants for a company or supplier (The following conflicts were disclosed) none

4. Stock or stock options in a company or supplier (The following conflicts were disclosed) Corin, Porosteon

5. Research support from a company or supplier as a Principal Investigator (The following conflicts were disclosed)

Apple, ZimmerBiomet

6. Other financial or material support from a company or supplier (The following conflicts were disclosed) none

7. Royalties, financial or material support from publishers (The following conflicts were disclosed) WoltersKluwer

8. Medical/Orthopaedic publications editorial/governing board (The following conflicts were disclosed) none

9. Board member/committee appointments for a society (The following conflicts were disclosed)

AAOS, AJRR, AAHKS, Knee Society, Hip Society

**Each author must sign AND print or type his/her name, date and submit a separate form**

In addition, one BLINDED Conflict of Interest form (no author names used) should be submitted per manuscript with all author disclosures.

James Huddleston
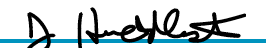
 Date. 11-19-23
