# Supplementary material for: Highlights of the 2023 American Joint Replacement Registry Annual Report
Source: Arthroplast Today. 2024 Feb 29;26:101325. doi: 10.1016/j.artd.2024.101325 (PMC11239969; doi:10.1016/j.artd.2024.101325)
Supplement: Conflict of Interest Statement for Ryan [file mmc4.pdf]

# INDIVIDUAL CONFLICT OF INTEREST STATEMENT

***American Association of Hip and Knee Surgeons***

(Adopted from the American Academy of Orthopaedic Surgeons disclosure statement)

The following form**must be filled out completely and submitted by each author (example, 6 authors, 6 forms). All items require a response. If there is no relevant disclosure for a given item, enter "*None.*"**

1. Royalties from a company or supplier (The following conflicts were disclosed)
None

2. Speakers bureau/paid presentations for a company or supplier (The following conflicts were disclosed)
None

3A. Paid employee for a company or supplier (The following conflicts were disclosed)
None

3B. Paid consultant for a company or supplier (The following conflicts were disclosed)
DePuy Synthes; Zimmer Biomet; Total Joint Orthopaedics

3C. Unpaid consultants for a company or supplier (The following conflicts were disclosed)
None

4. Stock or stock options in a company or supplier (The following conflicts were disclosed)
Azra Care

5. Research support from a company or supplier as a Principal Investigator (The following conflicts were disclosed)
Zimmer Biomet; Smith and Nephew

6. Other financial or material support from a company or supplier (The following conflicts were disclosed)
None

7. Royalties, financial or material support from publishers (The following conflicts were disclosed)
None

8. Medical/Orthopaedic publications editorial/governing board (The following conflicts were disclosed)

9. Board member/committee appointments for a society (The following conflicts were disclosed)

**Each author must sign AND print or type his/her name, date and submit a separate form**

In addition, one BLINDED Conflict of Interest form (no author names used) should be submitted per manuscript with all author disclosures.

| | | |
|---|---|---|
| Sean Ryan | | December 19, 2023 |
| Author Name (Print or Type) | Author Signature | Date |
